# Choosing the P.M.A. technique of Assisted Human Reproduction in male infertility – I.C.S.I.

**Published:** 2012

**Authors:** Gratiela Draghici

**Affiliations:** Emergency University Hospital, Bucharest

## I. PMA USED TECHNIQUES

A. The main techniques are the following:

1. In vivo fecundation - IUU=intrauterine insemination

   -GIFT=gametes intrafallopian transfer

2. In vitro fecundation -classic IVF

   -ICSI=intracytoplasmatic sperm injection

B. Annex techniques used in PMA:

1. Co-culture

2. Prolonged culture

3. Hatching techniques

4. Cryogenation

## II. CHOOSING THE PMA TECHNIQUE

Choosing the technique is done according to SMT – Sperm Migration Test, through which we can find out the available number of spermatozoos through fecundation, their quality, motility, and morphology.

Accordingly:

-if the number of spermatozoos/ml >20 million spermatozoos/ml,

with a motility of over 35-40% - we will choose IUI;

-if the number of spermatozoos is between 15 and 20 million/ml

Classic IVF is recommended;

-if the number of spermatozoos/ml<10 million/ml

ICSI is recommended.

Schematically, the results of the spermogram with the spermocytogramme, to which the spermatozoos migration-survival test is added, allows the appreciation of PMA type.

The exams must be repeated at least once at 3 months, ICSI being indicated if at least 10 million mobile spermatozoos exist after the migration test, or if the survival test at 17 hours is negative, or in cases of obstructive azoospermia, when the sperm is extracted through a testicular puncture-biopsy or open-biopsy.

The cause of the spermatic anomaly must be searched for through a clinical, biological, or hormonal examination, at the end of which the treatment is tried. In the absence of the therapeutic possibilities, the sperm complimentary examinations will allow the understanding of the infertility mechanism.

## III. ICSI INDICATIONS:

1. IVF failure;

2. Azoospermias;

3. Flagellar dyskinesias;

4. Oligoastenoteratospermia

5. Infertility due to a cervical cause;

6. Male autoimmunizations

In case of excretory azoospermia (through an obstacle on the seminal path), a seminal biochemical examination is recommended, together with FSH dosages, which are normal, urological, clinical and ultrasound explorations.

In the case of secretory azoospermia (associated with mucoviscidosis, with the atrophy of a testicle) the seminal biochemical examination is normal, the FSH level is high. Also in this case, clinical and ultrasound urological explorations are required.

In the case of flagellar diskinesia associated with anomalies of spermatozoos penetration of the cervical glera, the only useful exploration is represented by Huhner test. The test of cross penetration is used more rarely. In electronic microscopy, the study of the movement of spermatozoos can trace the absence of the external arms from the dineine or the sliding spermatozoos.

Antispermozoos autoimmunisations are met very rarely in females, and in males, they are highlighted by the interruption of the blood-testicle barrier, as a result of some external, surgical traumatisms or genital-urinary infections.

## IV. ICSI TECHNIQUE – TECHNICAL FACTORS WHICH ASSURE THE SUCCESS IN ICSI

1. Immobilization of the spermatozoos:

-by MECHANICAL SHOCK at the level of the flagellum – the partial sectioning of the flagellum with an injection micropipette, which allows the obtaining of a high fecundation rate, which can reach up to 15-30% and to 50-80%.

-by OSMOTIC SHOCK – through the incubation of the mobile spermatozoos in a hypo osmotic environment.

The “shocks” to which the spermatozoos are subjected before the microinjection, can develop disorganizations at the level of the plasmatic membrane, inducing the acrosome reaction and facilitating the drop of the spermatozoon in the oocyte and elaborating the cytosolic sperm factor, which is necessary in the process of oocyte activation [**1**].

That is why, even if, usually it is said that the sperm does not need special treatments in ICSI – a similar preparation to the IVF, the preparation of the spermatozoon which will be injected is indispensible.

2. Overcoming the plasmatic membrane

Due to the fact that the great plasticity of the plasmatic membrane allows the invagination without tearing apart during the intracytoplasmatic injection, the consistent aspiration of the citoplasma is required before the injection, which produces a higher number of major calcium peak (in case of light aspiration less calcium peaks are obtained).

## V. STUDY REGARDING THE ICSI APPLICABILITY IN MALE INFERTILITY

**1. THE PURPOSE OF THE STUDY –** the advantages of choosing ICSI as a PMA method in male infertility.

**2. STUDY GROUP –** 240 infertile couples

**3. RESULTS OF THE STUDY:**

=we have taken into consideration the patients whose sperm examination has demonstrated the physical, chemical and biological unsatisfactory result compared to the values accepted at an international scale, selecting a number of 22 couples out of 240;

=the 22 couples have been subjected to ICSI protocol, in these cases a number of 22 ovarian stimulations have been realized

=the ovarian stimulation has consisted of three stages:

-a-Decapeptyl inhibition 0,1 mg S.C. daily;

-b-hMG stimulation in doses of 4 f;

-c-triggering of the ovulation by using hCG 5000 U.I.

=in the case of 22 cycles of stimulation, a total number of 248 mature oocytes have been obtained, from which 223 intact oocytes have been selected;

=from the 223 intact oocytes only 116 oocytes have been fecundated through ICSI, representing 52% of the total of intact oocytes;

=from the total number of 116 fecundated oocytes, a number of 65 embryos have been transferred. Only the type A embryos in 4 cells stage have been transferred, the other type A and B embryos have been cryogenized (type C and D embryos do not resist to cryogenation);

=from the total number of 22 stimulated ovarian cycles, a number of 6 clinical pregnancies have been obtained.

**4. CONCLUSIONS**

=the result corresponds to a success rate of 33% for ICSI, a value net superior to the one obtained through classic IVF, which is of 17%;

=the results obtained are encouraging for the application of ICSI method in male infertility, having as a main purpose THE RAISE OF THE FECUNDABILITY OF THE OOCYTE, in cases of qualitative and/or quantitative anomalies of the sperm, thus raising the chances of obtaining a pregnancy;

= the start ICSI has taken, has revolutionized the treatment of male infertility and has allowed many males who were considered infertile to obtain a pregnancy in the couple, thus having only a few abnormal spermatozoos present in their sperm. Because in these cases, the spermatozoos cannot reach the ejaculation due to an irreparable obstruction, acquired or congenital, the sampling of spermatozoos upstream the obstacle or at the level of the testicle, is necessary [**2**].

The notion of epididimar maturation of the spermatozoos has been taken into consideration in the case of obtaining developmental pregnancies after the transfer in the uterus of the embryos obtained through classic IVF, when the spermatozoos coming from the level of the body or the epididimar head or at the level of the testicle are used. Despite the obtaining of some positive results, the enthusiasm initially generated by the use of IVF method has diminished. The failure of oocyte fecundation is unpredictable and without any apparent reasons no difference could be made between a fertilizing sperm and another that did not have this property. ICSI has allowed the solving of the problem to a great extent [**3**].
